# Supplementary material for: Splice-Junction-Based Mapping of Alternative Isoforms in the Human Proteome
Source: Cell Rep. Author manuscript; Available in PMC 2020 Jan 15. (PMC6961840; doi:10.1016/j.celrep.2019.11.026)
Supplement: 2 [file NIHMS1546469-supplement-2.zip › DF1/PXD010154/Lung/Lung_6_ATXN3_EEGSLCAQHCLNNLLQGEYFSPVELSSIAHQLDEEER.pdf]

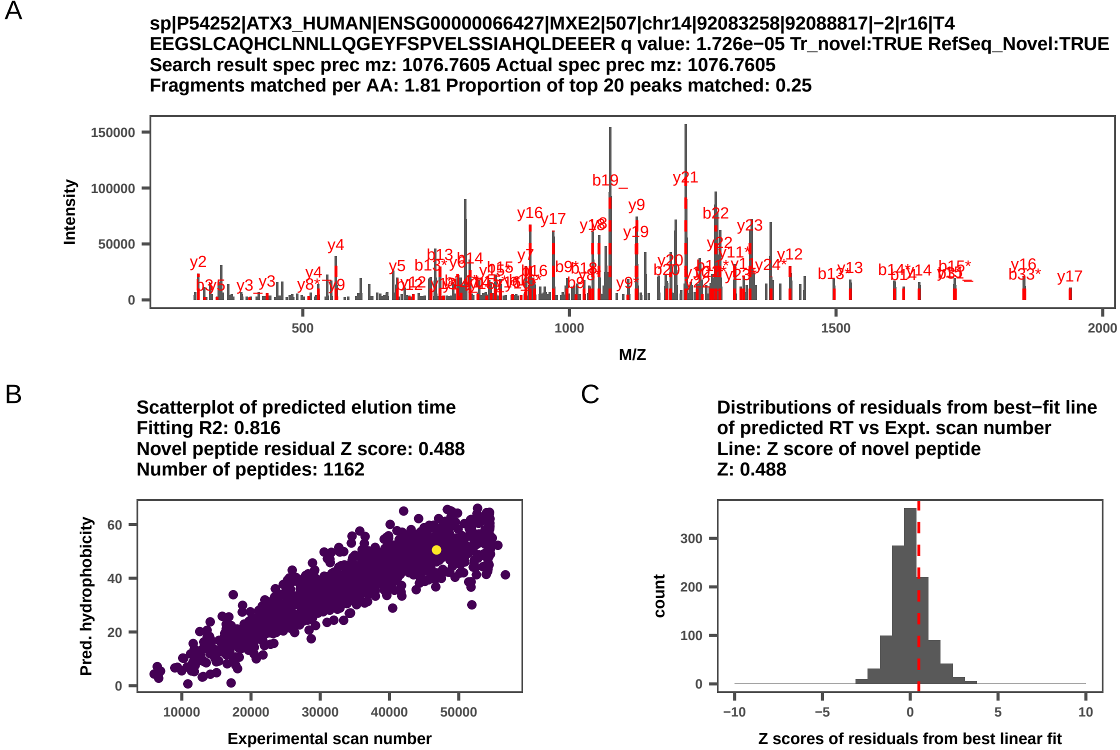
A
sp|P54252|ATX3_HUMAN|ENSG00000066427|MXE2|507|chr14|92083258|92088817|−2|r16|T4
EEGSLCAQHCLNNLLQGEYFSPVELSSIAHQLDEEER q value: 1.726e−05 Tr_novel:TRUE RefSeq_Novel:TRUE
Search result spec prec mz: 1076.7605 Actual spec prec mz: 1076.7605
Fragments matched per AA: 1.81 Proportion of top 20 peaks matched: 0.25
Intensity
150000
100000
50000
0
M/Z
500
1000
1500
2000
B
Scatterplot of predicted elution time
Fitting R2: 0.816
Novel peptide residual Z score: 0.488
Number of peptides: 1162
Pred. hydrophobicity
60
40
20
0
10000
20000
30000
40000
50000
Experimental scan number
C
Distributions of residuals from best−fit line
of predicted RT vs Expt. scan number
Line: Z score of novel peptide
Z: 0.488
count
300
200
100
0
−10
−5
0
5
10
Z scores of residuals from best linear fit
